# Predictors of occupational functioning in early-onset psychosis: a clinical study with register-based follow-up

**DOI:** 10.1192/j.eurpsy.2026.10543

**Published:** 2026-07-31

**Authors:** N. K. Andersen, B. Fagerlund, J. R. M. Jepsen, C. Hjorthøj, C. K. Lemvigh, J. Rydkjær, M. Melau, D. L. Vernal, A. K. Pagsberg

**Affiliations:** 1 Child and Adolescent Mental Health Center, Copenhagen University Hospital – Mental Health Services CPH; 2 Department of Clinical Medicine, University of Copenhagen; 3 Department of Psychology, University of Copenhagen; 4 Center for Neuropsychiatric Schizophrenia Research (CNSR), Mental Health Center, Glostrup, Copenhagen University Hospital – Mental Health Services CPH, Copenhagen; 5 Copenhagen Research Center for Mental Health, Mental Health Center Copenhagen, Copenhagen University Hospital, Mental Health Services-Capital Region of Denmark, Hellerup; 6 Department of Public Health, Section of Epidemiology, University of Copenhagen; 7 Mental Health Center Copenhagen, Copenhagen University Hospital – Mental Health Services CPH, Copenhagen; 8 Psychiatry, Aalborg University Hospital; 9 Department of Clinical Medicine, Aalborg University, Aalborg, Denmark

## Abstract

**Introduction:**

Knowledge on risk and protective factors for the long-term outcome of children and adolescents diagnosed with psychosis before age 18 (Early-Onset Psychosis, EOP) is essential to improve the outcomes in this patient group. Being in employment is a particular important outcome for individuals living with psychosis and for reducing the societal cost of illness. Identification of predictive markers for long-term occupational functioning can guide clinical practice and tailor treatment interventions to target modifiable predictors as well as strengthen the interventions for the patients at highest risk of poor outcomes.

**Objectives:**

To describe the long-term sociodemographic, functional, and clinical outcomes of EOP and healthy control (HC) participants, and explore predictors of occupational functioning.

**Methods:**

The study sample consists of 205 participants with EOP and 199 HCs aged between 10 and 17 at baseline. Clinical baseline data were linked to register-based outcome data from the Danish National Registers a mean of 11 years (range 4.5 to 23.5 years) after baseline assessment. At baseline, parental socioeconomic status, psychosis symptoms, neurocognitive functioning, social functioning, and drug use were assessed. At follow-up, register-data on employment status, marital status, income, educational level, supported housing, psychiatric hospitalizations, somatic hospitalizations and deaths were retrieved, and compared for EOP and HC participants. Predictors of competitive employment were explored using univariable and multivariable logistic regression.

**Results:**

At follow-up participants were between 18 and 41 years of age. Register-based outcomes showed markedly poorer sociodemographic, functional, and clinical outcomes for individuals with EOP compared to HCs. At follow-up 47% of EOP and 94% of HC participants were in competitive employment. Being in competitive employment at follow-up was in univariable analyses predicted by higher household income and better neurocognitive functioning (estimated IQ; verbal fluency; and attention and information processing speed) at baseline. These baseline measures did not remain significantly associated with competitive employment in multivariate analyses.

**Conclusions:**

The finding that almost half of individuals with EOP was in employment at follow-up is encouraging compared to previous literature. The study nonetheless emphasizes the importance in continuous efforts to improve outcomes in early-onset psychosis, as outcomes across sociodemographic, functional, and clinical outcomes remains significantly poorer than controls. The study points to neurocognitive functioning as a potential modifiable treatment target to improve occupational functioning.

**Disclosure of Interest:**

None Declared

